# Supplementary material for: Clinical characteristics of Japanese patients with chronic obstructive pulmonary disease (COPD) with comorbid interstitial lung abnormalities: A cross-sectional study
Source: PLoS One. 2020 Nov 10;15(11):e0239764. doi: 10.1371/journal.pone.0239764 (PMC7654824; doi:10.1371/journal.pone.0239764)
Supplement: Attachment — Submitted filename: Response to Reviewers_Manabu Ono.docx [file pone.0239764.s001.docx]

July 16, 2020

Dr Muhammad Adrish

Academic Editor
PLOS ONE

Re: PONE-D-20-07036, Clinical characteristics of Japanese patients with chronic obstructive pulmonary disease (COPD) with comorbid interstitial lung abnormalities: A cross-sectional study

Dear Dr Muhammad Adrish,

We would like to thank you and the reviewers for the helpful comments on the original version of our manuscript. We have taken all these comments into account and resubmit a revised version of our paper.

In the responses to the comments of reviewers, we have included a point-by-point list of responses on separate pages.

We hope that the revised version of our paper is now suitable for publication in PLOS ONE and we look forward to hearing from you.

Yours sincerely,

Manabu Ono

Department of Respiratory Medicine

Japanese Red Cross Ishinomaki Hospital,

71 Nishimichishita, Hebita, Ishinomaki 986-8522, Japan.

Phone: +81-225-21-7220, fax: +81-225-96-0122

E-mail: [ono_manabu@rm.med.tohoku.ac.jp](mailto:ono_manabu@rm.med.tohoku.ac.jp)

**Responses to comments from the Academic Editor**

We would like to thank the Editor for the comments and useful suggestions. We have taken all these comments into account and resubmit a revised version of our paper.

*Comment*

Journal Requirements:

When submitting your revision, we need you to address these additional requirements.

1. Please ensure that your manuscript meets PLOS ONE's style requirements, including those for file naming. The PLOS ONE style templates can be found at

https://journals.plos.org/plosone/s/file?id=wjVg/PLOSOne_formatting_sample_main_body.pdf and https://journals.plos.org/plosone/s/file?id=ba62/PLOSOne_formatting_sample_title_authors_affiliations.pdf

*Response*

We have corrected our manuscript so as to meet PLOS ONE's style requirements.

*Comment*

2. In your Methods section, please provide additional information about the participant recruitment method and the demographic details of your participants.

Please ensure you have provided sufficient details to replicate the analyses such as:

a) the recruitment date range (month and year),
b) a description of how participants were recruited, and
c) descriptions of where participants were recruited and where the research took place.

*Response*

Thank you very much for your helpful comments. Upon your suggestion, we described in more detail about the participant recruitment method and the demographics of the participants in the Method section (page 6, paragraph 4, line 2-5).

*Comment*

3. Please provide a sample size and power calculation in the Methods, or discuss the reasons for not performing one before study initiation.

*Response*

The sample size and power calculation were not performed because this is an observational study, not an intervention study.

*Comment*

4. Please include additional information regarding the COPD Assessment Test questionnaire used in the study and ensure that you have provided sufficient details that others could replicate the analyses. For instance, if you developed a questionnaire as part of this study and it is not under a copyright more restrictive than CC-BY, please include a copy, in both the original language and English, as Supporting Information.

*Response*

International and national guidelines state that qualities of life of patients with COPD can be assessed using the COPD Assessment Test (CAT) (ref. Global Initiative for Chronic Obstructive Lung Disease. Global Strategy for the Diagnosis, Management and Prevention of COPD, updated 2020; The Japanese Respiratory Society Guidelines for the Diagnosis and Treatment of COPD, the 5th Edition. 2018). The CAT was originally described in English, and validated Japanese translation version is available (ref. Respir Investig 2012;50:34–39).

*Comment*

5. We note that you have indicated that data from this study are available upon request. PLOS only allows data to be available upon request if there are legal or ethical restrictions on sharing data publicly. For information on unacceptable data access restrictions, please see <http://journals.plos.org/plosone/s/data-availability#loc-unacceptable-data-access-restrictions>.

In your revised cover letter, please address the following prompts:

a) If there are ethical or legal restrictions on sharing a de-identified data set, please explain them in detail (e.g., data contain potentially identifying or sensitive patient information) and who has imposed them (e.g., an ethics committee). Please also provide contact information for a data access committee, ethics committee, or other institutional body to which data requests may be sent.

b) If there are no restrictions, please upload the minimal anonymized data set necessary to replicate your study findings as either Supporting Information files or to a stable, public repository and provide us with the relevant URLs, DOIs, or accession numbers. Please see http://www.bmj.com/content/340/bmj.c181.long for guidelines on how to de-identify and prepare clinical data for publication. For a list of acceptable repositories, please see http://journals.plos.org/plosone/s/data-availability#loc-recommended-repositories.

We will update your Data Availability statement on your behalf to reflect the information you provide.

*Response*

Due to the ethical restriction of the ICON registry, supporting data is not available.

*Comment*

6. Please amend your list of authors on the manuscript to ensure that each author is linked to an affiliation. Authors' affiliations should reflect the institution where the work was done (if authors moved subsequently, you can also list the new affiliation stating "current affiliation:…." as necessary).

*Response*

One author moved subsequently. We rewrote the list of affiliations in the manuscript.

**Responses to comments from Reviewer #1**

We would like to thank Reviewers #1 for the critical comments and useful suggestions that have helped us to improve our paper. As indicated in the responses that follow, we have taken all the comments and suggestions in the revised version of our paper.

*Comment*

Reviewers' comments:

Reviewer's Responses to Questions

Comments to the Author

1. Is the manuscript technically sound, and do the data support the conclusions?

The manuscript must describe a technically sound piece of scientific research with data that supports the conclusions. Experiments must have been conducted rigorously, with appropriate controls, replication, and sample sizes. The conclusions must be drawn appropriately based on the data presented.

Reviewer #1: Yes

2. Has the statistical analysis been performed appropriately and rigorously?

Reviewer #1: Yes

3. Have the authors made all data underlying the findings in their manuscript fully available?

The PLOS Data policy requires authors to make all data underlying the findings described in their manuscript fully available without restriction, with rare exception (please refer to the Data Availability Statement in the manuscript PDF file). The data should be provided as part of the manuscript or its supporting information, or deposited to a public repository. For example, in addition to summary statistics, the data points behind means, medians and variance measures should be available. If there are restrictions on publicly sharing data-e.g. participant privacy or use of data from a third party-those must be specified.

Reviewer #1: Yes

4. Is the manuscript presented in an intelligible fashion and written in standard English?

PLOS ONE does not copyedit accepted manuscripts, so the language in submitted articles must be clear, correct, and unambiguous. Any typographical or grammatical errors should be corrected at revision, so please note any specific errors here.

Reviewer #1: Yes

*Comment*

5. Review Comments to the Author

Please use the space provided to explain your answers to the questions above. You may also include additional comments for the author, including concerns about dual publication, research ethics, or publication ethics. (Please upload your review as an attachment if it exceeds 20,000 characters)

Reviewer #1: An interesting manuscript in its field, indeed I agree with the authors, however; do they suggest a method that these lesions can be differentiated. Can we use some kind of vehicle that binds differently with each interstitial lesion?

*Response*

Thank you for your helpful and constructive comments. We differentiate ILAs from pulmonary fibrosis in terms of existence of honey-comb-like lesions. The radiologic features of ILAs consist of interstitial fibrosis adjacent to a bronchiole with minimal associated emphysema, which is different from honey-comb lesion in pulmonary fibrosis based upon the previous report (Am J Respir Crit Care Med 2018;198:e44–e68). In the Materials and Method section we described in more detail the evaluation of ILAs (page 8,paragraph 5, line 1-9)

*Comment*

6. PLOS authors have the option to publish the peer review history of their article (what does this mean?). If published, this will include your full peer review and any attached files.

If you choose "no", your identity will remain anonymous but your review may still be made public.

Do you want your identity to be public for this peer review? For information about this choice, including consent withdrawal, please see our Privacy Policy.

Reviewer #1: No

**Responses to comments from Reviewer #2**

We would like to thank Reviewers #2 for the critical comments and useful suggestions that have helped us to improve our paper.

*Comment*

Reviewers' comments:

Reviewer's Responses to Questions

Comments to the Author

1. Is the manuscript technically sound, and do the data support the conclusions?

The manuscript must describe a technically sound piece of scientific research with data that supports the conclusions. Experiments must have been conducted rigorously, with appropriate controls, replication, and sample sizes. The conclusions must be drawn appropriately based on the data presented.

Reviewer #2: Partly

*Response*

As indicated in the responses that follow, we have taken all the comments and suggestions in the revised version of our paper.

*Comment*

2. Has the statistical analysis been performed appropriately and rigorously?

Reviewer #2: Yes

*Response*

We wish to thank you for your helpful comments.

*Comment*

3. Have the authors made all data underlying the findings in their manuscript fully available?

The PLOS Data policy requires authors to make all data underlying the findings described in their manuscript fully available without restriction, with rare exception (please refer to the Data Availability Statement in the manuscript PDF file). The data should be provided as part of the manuscript or its supporting information, or deposited to a public repository. For example, in addition to summary statistics, the data points behind means, medians and variance measures should be available. If there are restrictions on publicly sharing data-e.g. participant privacy or use of data from a third party-those must be specified.

Reviewer #2: No

*Response*

Due to the ethical restriction of the ICON registry, supporting data is not available.

*Comment*

4. Is the manuscript presented in an intelligible fashion and written in standard English?

PLOS ONE does not copyedit accepted manuscripts, so the language in submitted articles must be clear, correct, and unambiguous. Any typographical or grammatical errors should be corrected at revision, so please note any specific errors here.

Reviewer #2: Yes

*Comment*

5. Review Comments to the Author

Please use the space provided to explain your answers to the questions above. You may also include additional comments for the author, including concerns about dual publication, research ethics, or publication ethics. (Please upload your review as an attachment if it exceeds 20,000 characters)

Reviewer #2: Major points:

1. There is a need for more clarity in the paper. Are the authors trying to define the incidence of new ILA in COPD, or study its progression? They mention 'the frequency of newly appeared ILAs during the observational period in the patients with COPD,' but at the same time also use progression as a descriptive term. These are 2 different aspects, and it is not clear of serial CT's/PFT's were done to define progression. This is major aspect which needs clarification.

*Response*

Thank you very much for your helpful comments. In this study we evaluate the incidence of new ILAs, but did not focus on the development of ILAs.

*Comment*

2. They mention clinical characteristics, which is exacerbation frequency in COPD/ILA vs COPD only. What is the clinical significance of this exacerbation frequency in this context - with the low number with ILA, it is difficult to comment on this, and has limited clinical significance. In addition, the word clinical characteristics implies more clinical features, and may be too broad a term for what here is a specific analysis of exacerbations.

*Response*

The possibility of bias arises because of the low number with ILA. Even in case of small study samples, propensity score matching can yield correct estimations (BMC Med Res Methodol. 2012 May 30;12:70.). Therefore, we analysed data with propensity score matching to control for selection bias. Upon your suggestion, we added the clinical importance of COPD exacerbation in Discussion section.

*Comment*

3. The mean age of these patients is 72.9 years, which is also the age for rising ILD prevalence. The fundamentally question is that is this an incidental abnormality arising in this elderly cohort, and is there any link at all with COPD - an epiphenomenon at best? What are the pathophysiological correlates which make such a relationship tenable, above and beyond the link with smoking irritants? Is it just part of a continuum, or is post-infectious scarring? Please comment on this.

*Response*

We appreciate your helpful suggestion. We rewrote the Discussion section.

*Comment*

4. The significance of these CT ILA types are not clear, is GGO or reticulation different from honeycombing? The true impact of these findings is not clear.

*Response*

As described in the Method section, ILAs were defined as nondependent changes affecting more than 5% of any lung zone and included nondependent ground-glass or reticular abnormalities, diffuse centrilobular nodularity, nonemphysematous cysts, honeycombing, and traction bronchiectasis according to the previous study (ref. N Engle J Med 2011;364:897–906).

*Comment*

Minor points

1. The exacerbations were based on patient interviews, diaries, and record review, and need for drugs, and may not reflect a true exacerbation.

*Response*

We obtained exacerbation information requiring use of antibiotics and/or systemic corticosteroids and hospitalization from direct patients or caregivers interview, self-management diaries, referral letters from general practitioners and medical records review. This evaluation system was reported previously (Int J Chron Obstruct Pulmon Dis 2018;13:3947-3955).

*Comment*

6. PLOS authors have the option to publish the peer review history of their article (what does this mean?). If published, this will include your full peer review and any attached files.

If you choose "no", your identity will remain anonymous but your review may still be made public.

Do you want your identity to be public for this peer review? For information about this choice, including consent withdrawal, please see our Privacy Policy.

Reviewer #2: No
